# Smart and Mechanically Enhanced Zein–Gelatin Films Incorporating Cellulose Nanocrystals and Alizarin for Fish Spoilage Monitoring

**DOI:** 10.3390/foods14173015

**Published:** 2025-08-28

**Authors:** Leonardo Sentanin, Josemar Gonçalves de Oliveira Filho, Mariana Buranelo Egea, Luiz Henrique Capparelli Mattoso

**Affiliations:** 1 Embrapa Instrumentation, Brazilian Agricultural Research Corporation, 15 de Novembro Street, 1452, Centro, São Carlos 13561-206, SP, Brazil; leonardo.sentanin@gmail.com (L.S.); luiz.mattoso@embrapa.br (L.H.C.M.); 2 Goiano Federal Institute of Education, Science and Technology, Campus Rio Verde, Highway BR 452, Km 2, Rural Zone, Rio Verde 75901-970, GO, Brazil; mariana.egea@ifgoiano.edu.br

**Keywords:** smart films, natural pigments, food stability

## Abstract

The shelf life of perishable foods is traditionally determined by microbiological, chemical, and sensory analyses, which are well-established and reliable. However, these methods can be time-consuming and resource-intensive, and they may not fully account for unexpected storage deviations, such as temperature fluctuations or equipment failures. Smart films emerge as a promising alternative, enabling rapid, visual, and low-cost food quality monitoring. This study developed smart films based on zein/gelatin/cellulose nanocrystals (Z/G/CNC) functionalized with alizarin (AL, 0–3% *w*/*w*), produced by casting (12.5% zein, 12.5% gelatin, and 5% CNC *w*/*w*). The films were characterized for morphological, physicochemical, thermal, and spectroscopic properties, chromatic response at pH 3–11, activity against *Escherichia coli* and *Staphylococcus aureus*, and applicability in monitoring Merluccid hake fillets. The incorporation of AL reduced water solubility, increased water vapor permeability and contact angle, imparted a more intense orange coloration, and improved thermal resistance. AL also increased thickness and elongation at break while reducing tensile strength and Young’s modulus. All films exhibited excellent UV-blocking capacity (<1% transmittance). Noticeable color changes were observed, with the Z/G/CNC/AL1 film being the most sensitive to pH variations. During Merluccid hake storage, ΔE values exceeded 3 within 72 h, with a color change from orange to purple, correlating with fillet pH (8.14) and total volatile basic nitrogen (TVB-N) (24.73 mg/100 g). These findings demonstrate the potential of the developed films as biodegradable sensors for smart packaging of perishable foods.

## 1. Introduction

The conventional estimation of food shelf life relies on microbiological, chemical, and sensory analyses performed under standardized storage conditions. However, these estimations may not fully capture the effects of deviations from these conditions. For example, unsuitable storage, such as accidental refrigeration interruptions, can accelerate spoilage and pose health risks to consumers, while foods still suitable for consumption may be discarded prematurely, leading to economic losses and impacting food security [1]. Conventional methods are also costly and time-consuming, which has driven interest in low-cost and straightforward colorimetric approaches for real-time quality monitoring [2].

Smart films, especially pH-sensitive ones, have gained attention for monitoring microbial and chemical changes during storage. These films contain interactive indicators that respond to temperature, pH, gases, or microbial metabolites, showing visible color changes that signal spoilage [3,4].

Biopolymer-based films are attractive for sustainable packaging, with gelatin and zein standing out due to their natural, renewable, and biodegradable characteristics. Gelatin (G) offers excellent film-forming properties but suffers from poor mechanical strength and high moisture sensitivity [5]. Combining gelatin with zein (Z), a hydrophobic corn protein, improves water resistance and barrier properties through hydrophobic and hydrogen-bond interactions [6,7].

To further enhance mechanical performance, cellulose nanocrystals (CNCs) have been explored as nanoreinforcements due to their high aspect ratio, crystallinity (~90%), elastic modulus (~150 GPa), and tensile strength (~7.5 GPa) [8,9]. Leite et al. [10] reported a 64% increase in the tensile strength of gelatin films with only 4% CNCs.

Traditionally, synthetic indicators such as bromocresol purple and bromophenol blue have been used in smart packaging. However, their potential toxicity and impact on food sensory properties limit their application [11]. Thus, natural, non-toxic, and biodegradable pigments have gained attention as safer alternatives [1]. Among them, alizarin (AL) (1,2-dihydroxyanthraquinone) stands out for its high pH sensitivity. Its color change results from structural ionization: yellow in acidic conditions (pH 2–4, neutral molecules), red at near-neutral pH (pH 5–7, monoanionic species), and purple in alkaline conditions (pH 9–12, anionic species) [12]. This behavior makes alizarin a promising natural indicator for smart food packaging.

Therefore, this study aimed to develop Z/G/CNC smart films functionalized with AL and evaluate their potential for monitoring food quality.

## 2. Materials and Methods

### 2.1. Materials

Gelatin (Type B, Bloom 250, MW ~ 100 kDa), corn zein (grade Z3625, 22–24 kDa), and AL were purchased from Sigma Chemical Co. (St. Louis, MO, USA). Acetic acid (analytical reagent grade) was obtained from Dinamica (Campo Grande, Brazil). All other reagents were of analytical grade. Microbial strains of *Escherichia coli* (ATCC 25922) and *Staphylococcus aureus* (ATCC 25923) were obtained from the collection of Embrapa Instrumentation (São Carlos, Brazil). The CNCs, which were obtained from Bionano (São Carlos, SP, Brazil). CNCs had an average length of 384 ± 102 nm and a diameter of 15 ± 2 nm (resulting in an aspect ratio of approximately 25.6) and a zeta potential value of –22.62 mV. Merluccid hake (*Merluccius hubbsi*) fillets were obtained packaged and refrigerated from a local supermarket in São Carlos, Brazil.

### 2.2. pH-Sensitivities of AL

The pH sensitivities of AL were assessed by dissolving 10 mg of AL in 5 mL of various buffer solutions (pH 2–11) for 3 min. The solution was then transferred to a small sample vial. The UV-visible absorption spectra of AL were recorded in the abovementioned buffer solutions using a UV-Vis spectrophotometer (Shimadzu Corporation, Kyoto, Japan) in the 450–800 nm wavelength range.

### 2.3. Production of Smart Films

The smart films were prepared by dissolving gelatin (G) (12.5% *w*/*v*), zein (Z) (12.5% *w*/*v*), glycerol (10% *w*/*w* based on polymer), and AL at concentrations of 1, 2, and 3% (*w*/*w* of biopolymer) in 16 mL of acetic acid, using an Erlenmeyer flask wrapped with light-proof paper. These proportions were defined based on preliminary experiments aimed at achieving an optimal dispersion of the components and satisfactory film-forming properties. The mixtures were stirred with a magnetic stirrer for 12 h to ensure complete dissolution. After dissolution, the casting technique was employed by pouring the solution into small Petri dishes (90 mm × 10 mm), controlling the average wet thickness of the films. Finally, the wet solutions were dried in a forced-air oven (Marconi MA035/2, Piracicaba, SP, Brazil) at 35 °C for 48 h. The treatments were designated as Z/G/CNC (control, without alizarin) and Z/G/CNC/AL1, Z/G/CNC/AL2, and Z/G/CNC/AL3, corresponding to the incorporation of 1%, 2%, and 3% (*w*/*w*) alizarin, respectively.

### 2.4. Characterization of Smart Films

Scanning electron microscopy (SEM) was used to analyze the films’ morphology. Samples (5 × 5 mm^2^) were mounted on aluminum stubs, sputter-coated with gold–palladium (Denton Desk II, Denton Vacuum, Moorestown, NJ, USA), and imaged using a JEOL JSM-6510 microscope (JEOL Ltd., Tokyo, Japan) at 5 kV under ambient conditions.

Water solubility (WS) was determined following Kavoosi et al. [13] with modifications. Film samples (2 cm^2^) were dried at 100 ± 5 °C for 24 h (initial dry weight), immersed in 50 mL distilled water at 23 ± 2 °C for 24 h, and re-dried under the same conditions to obtain the final dry weight. WS (%) was calculated as in Equation (1).

(1) $$Water\;solubility(\%)=\frac{initial\;dry\;weight-final\;dry\;weight}{initial\;dry\;weight}*100$$

Water vapor permeability (WVP) was measured according to ASTM E96/E96M-16 [14] using PTFE permeation cells (24 mm diameter, 10 mm height) filled with 2 mL distilled water (100% RH). Experiments were performed at 25 °C and 50% RH in a climatic chamber (Ethik 420–2TS, Vargem Grande Paulista, SP, Brazil), with eight replicates over 24 h. WVP was calculated as in Equation (2).

(2) $$WVP=\frac{m}{t}.\frac{x}{A.\Delta p}$$

where WVP is the water vapor permeability (g/ms Pa) calculated from the variation in mass (m) due to the loss of water vapor that crossed a polymeric material with width (x) and area (A) during the time interval (t) under pressure (Δp) (the difference between the water-vapor pressures of the external and internal media).

Contact angle was evaluated by the sessile drop method (Theta Lite optical tensiometer, Attension, Biolin Scientific, Gothenburg, Sweden). A 3-μL water droplet was placed on the film surface, and images were recorded every 1 s for 1 min usingOneAttension software (version 2.4). Ten replicates were analyzed per sample.

Color analysis was performed with a Konica Minolta CM-5 colorimeter (Minolta, Tokyo, Japan) in the CIE Lab* system. Hue angle (h°), chroma (C), and total color difference (ΔE) were calculated using Equations (3)–(5), respectively.

(3) h° = tan^−1^ (b*/a*)

(4) C* = (a*^2^ + b*^2^)1/2

(5) $$\Delta E=\sqrt{(L^{*}-L{)}^{2}+(a^{*}-a{)}^{2}+(b^{*}-b{)}^{2}}$$

Light transmittance (250–800 nm) was measured by UV-Vis spectrophotometry (Lambda 750, PerkinElmer, Waltham, MA, USA).

Thickness was measured at ten random points with a micrometer (MDC-25, Mitutoyo, Tokyo, Japan). Tensile tests followed ASTM D882-97 using a TA.XT Plus texture analyzer (Stable Micro Systems, Godalming, UK) with a 50-N load cell, grip separation 20 mm, and crosshead speed 80 mm/min. Maximum tensile strength (MPa) and elongation at break (%) were determined from stress–strain curves.

Thermal stability was assessed by TGA (Q500, TA Instruments, New Castle, DE, USA) from 50 to 600 °C at 10 °C/min. FTIR spectra were obtained (Cary 630, Agilent Technologies, Santa Clara, CA, USA) using ATR mode (4000–400 cm^−1^, 32 scans, 4 cm^−1^ resolution). X-ray diffraction (XRD; X’Pert Pro DRX-6000, Shimadzu, Kyoto, Japan) was performed with Cu Kα radiation (40 kV, 35 mA) in the 5–70° 2θ range at 2°/min.

### 2.5. Color Response of Smart Films Under Different pH Conditions

The color responses of the smart films to pH changes were evaluated using a HunterLab colorimeter through CIELab color parameters. Film sections (2 cm^2^) were immersed in 10 mL buffer solutions with pH values ranging from 3 to 10, and color parameters were recorded [15].

### 2.6. Microbial Response of Smart Films

The microbial response of the films was tested against *Escherichia coli* and *Staphylococcus aureus* using a modified agar diffusion method [16]. A single bacterial colony was cultured in sterile broth, incubated at 37 °C for 24 h, and diluted to a turbidity of 0.5 McFarland. The suspension was spread evenly over agar plates with sterile swabs and dried for 10 min. Film samples (1 × 1 cm^2^; three replicates per treatment) were placed on the agar surface and incubated at 37 °C for 24 h. After incubation, films were visually examined for color changes, and antibacterial activity was assessed by observing inhibition zones around the samples.

### 2.7. Application of Smart Films to Monitor Fish Fillet Freshness

The ability of the smart films to monitor the freshness of *Merluccius hubbsi* (Merluccid hake) fillets was evaluated following a modified version of Gasti et al. [17]. Film samples (3 × 3 cm^2^) were placed directly on ~80 g fillets, which were stored in 150 × 150 × 18 mm^3^ expanded-polystyrene trays covered with polyvinyl chloride film at 25 °C for 72 h. Color changes were recorded photographically, and total color difference (ΔE) was calculated (Equation (5)). Simultaneously, fillet pH was measured using a digital pH meter (PHS-3B, INESA Scientific Instrument Co., Ltd., Shanghai, China) following Zhai et al. [18]; Total Volatile Basic Nitrogen (TVB-N) was determined according to Zhai et al. [18], and Total Viable Count (TVC) was assessed following the Chinese standard GB4789.2-2022.

### 2.8. Statistical Analysis

All results were expressed as mean ± standard deviation. Statistical differences were determined by Tukey’s test, with significance set at *p* < 0.05.

## 3. Results

### 3.1. Film Characterization

**Figure 1:**
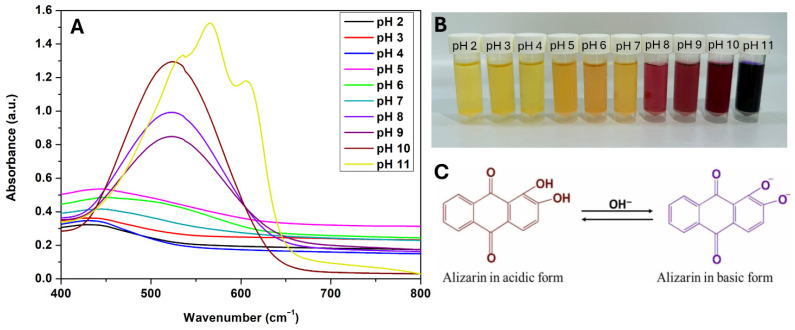
UV–vis absorption spectra of alizarin at pH 2–11 (**A**); visible color changes of alizarin solution at pH 2–11 (**B**); structural transformations of alizarin associated with color changes at different pH values (**C**).

The UV–vis absorption spectrum and the color change of AL solution at different pH levels are shown in Figure 1. As illustrated in Figure 1B, AL displays a visible color shift from yellow in acidic conditions (pH 2–4), through orange (pH 5–7) and purple (pH 8–10), to blue at pH 11. According to the spectrum in Figure 1A, this color transition corresponds to a bathochromic shift of the maximum absorption peak from 430 nm (acidic) to 540 nm (alkaline). This behavior results from the structural conversion of AL between its neutral, monoanionic, and dianionic forms as pH increases, as depicted in Figure 1C, which outlines the chemical transformations responsible for the observed shift in light absorption and, consequently, solution color [12,19].

**Figure 2:**
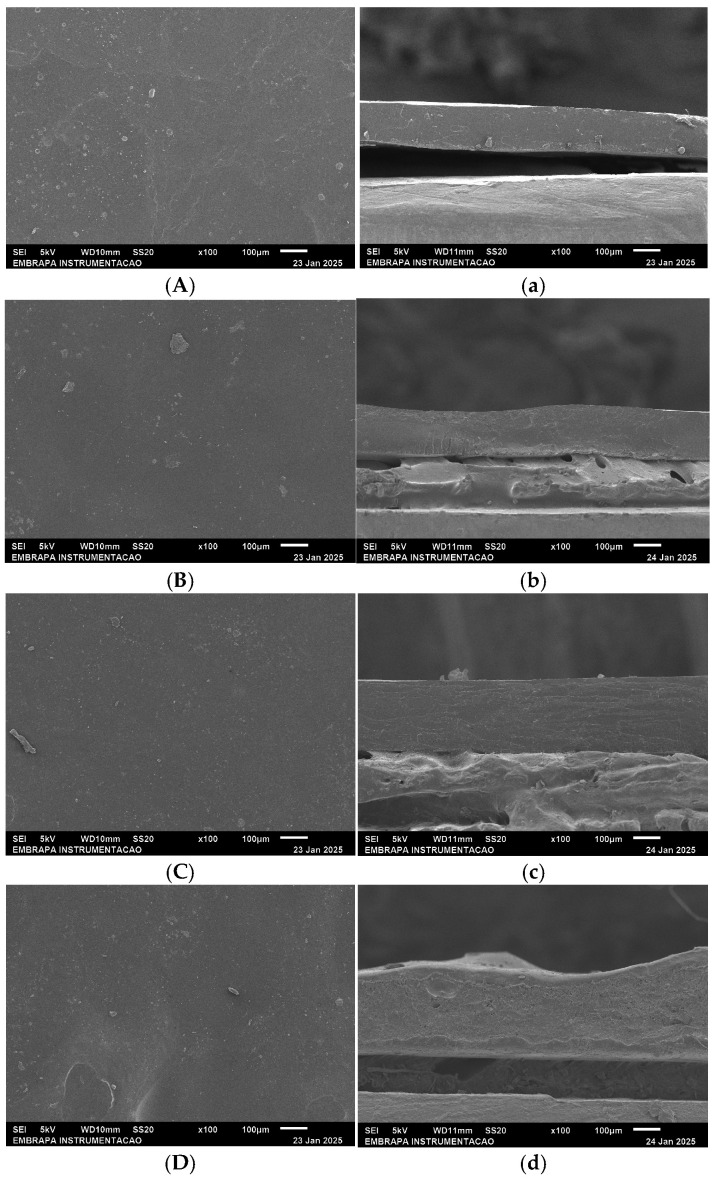
Scanning electron micrographs of nanocomposite films based on zein (Z), gelatin (G), and cellulose nanocrystals (CNC) functionalized with alizarin (AL). (**A**–**D**) Surface morphology image of Z/G/CNC, Z/G/CNC/AL1, Z/G/CNC/AL2, and Z/G/CNC/AL3, respectively, and (**a**–**d**) fracture surface image of Z/G/CNC, Z/G/CNC/AL1, Z/G/CNC/AL2, and Z/G/CNC/AL3, respectively.

SEM micrographs (Figure 2) reveal distinct morphological and structural changes in Z/G/CNC films with increasing AL content (1, 2, and 3%). The control film (Z/G/CNC, Figure 2A) exhibits a relatively homogeneous surface with small, well-dispersed aggregates. Its cross-section (Figure 2a) shows a compact, well-defined structure, suggesting good compatibility between the polymer matrix and nanocellulose.

In contrast, the surface of Z/G/CNC/AL1 (Figure 2B) displays greater heterogeneity with visible agglomerates, indicating poor dispersion or limited interaction between AL and the matrix. Its cross-section (Figure 2b) reveals a more porous and less uniform structure, suggesting structural disruption caused by AL. Similarly, Z/G/CNC/AL2 (Figure 2C) shows surface irregularities with scattered large agglomerates. The corresponding cross-section (Figure 2c) exhibits a stratified internal structure, possibly resulting from phase separation induced by higher AL concentration.

At 3% alizarin (Z/G/CNC/AL3), the film presents an even more heterogeneous surface with a greater number of large aggregates. The cross-section (Figure 2d) reveals surface deformities and a disorganized internal morphology, likely due to matrix destabilization, which may compromise the film’s mechanical integrity.

Overall, increasing AL concentration leads to the formation of larger aggregates due to intensified intermolecular interactions, which can negatively affect the mechanical performance of the films [20]. A similar behavior was reported by [21] in konjac glucomannan and polyvinyl alcohol films. The addition of AL to the films increased heterogeneity and roughness.

Table 1 presents the results of the water-related properties of the smart films. The water solubility of the Z/G/CNC-based films decreased significantly from 80.25% (control) to 60.73–64.09% upon incorporation of AL. This reduction is attributed to the hydrophobic nature of AL, which arises from its anthracene core with two carbonyl groups. The anthracene backbone is a conjugated system of benzene rings, predominantly nonpolar and poorly soluble in water. Although the presence of carbonyl groups (C=O) introduces some polarity, it is insufficient to render the molecule highly hydrophilic [20]. A similar behavior was reported by Lin et al. [22] in curcumin–β-cyclodextrin and cinnamaldehyde films when AL was incorporated into the matrix at a 1% concentration.

On the other hand, the films’ water vapor permeability (WVP) increased significantly with the incorporation of AL (Table 1), compared to the control film. This effect was most pronounced in Z/G/CNC/AL1, which exhibited the highest WVP value. This can be attributed to the low AL concentration, which led to the formation of isolated aggregates that were insufficient to establish an effective hydrophobic barrier, thereby favoring moisture absorption and increased permeability. SEM analysis (Figure 2) supports this finding, revealing a less uniform structure with visible aggregates on the surface.

In contrast, films with higher AL concentrations (Z/G/CNC/AL2 and AL3) showed WVP values closer to the control. This may be due to the formation of more abundant hydrophobic microdomains within the matrix, visible in the SEM images as dispersed surface aggregates, which likely act as physical barriers to water vapor transmission. This behavior is consistent with the chemical structure of AL, whose anthracene ring and carbonyl groups contribute to its hydrophobic character [19].

Contact angle measurement is a key parameter in evaluating films for food packaging applications, as the material’s hydrophobicity directly affects its effectiveness as a moisture barrier. Hydrophobic materials are particularly desirable in this context, in addition to being non-toxic [23].

As shown in Table 1, the incorporation of AL increased the contact angle values of the Z/G/CNC films, indicating enhanced surface hydrophobicity due to the presence of the dye. This result is consistent with the reduced water solubility observed in the films containing AL. A similar behavior was reported by Xia, Ma, Ma, Yao, Xu, Ji, and Zhang [20] in cellulose-based films functionalized with AL.

Table 2 presents the chromatic parameters of the different films, focusing on color coordinates (L*, h°, and C*) and total color difference (ΔE*). Incorporating AL into the Z/G/CNC films decreased lightness (L*), from 87.39 in the control film to a range between 57.03 and 71.30, indicating a darker appearance. Similarly, the hue angle (h°) decreased from 93.91 to 76.31 and 55.01, reflecting a color shift from yellow to orange as the AL concentration increased. The chroma values (C*) increased with the addition of the dye, indicating greater color saturation (Table 2). Furthermore, the total color difference (ΔE*) increased with AL incorporation, confirming that the film’s color changes are visually perceptible.

Overall, adding AL resulted in darker films with an orange hue and more intense coloration. Similar findings were reported by Zou et al. [24] in polycaprolactone (PCL) nanofiber films containing AL.

**Figure 3:**
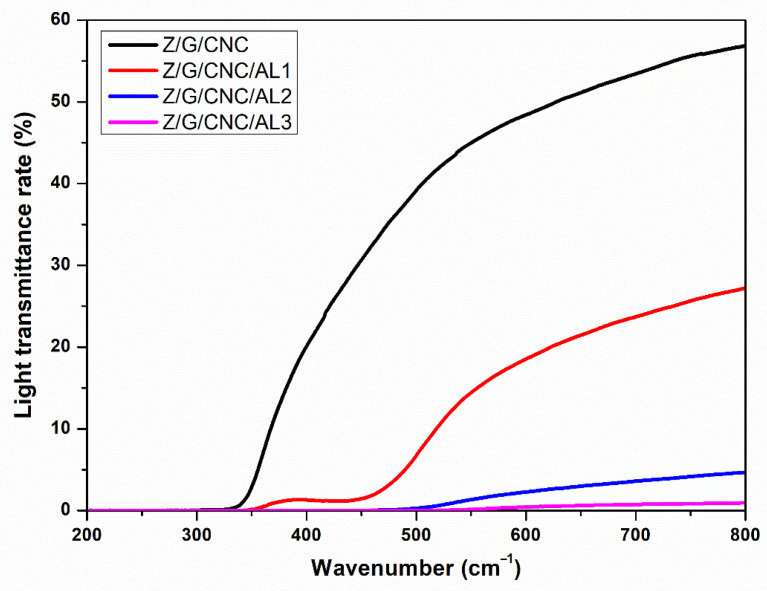
Light transmittance curves of nanocomposite films based on zein (Z), gelatin (G), and cellulose nanocrystals (CNC) functionalized with alizarin (AL).

Figure 3 shows the films’ UV/vis light transmittance. All films exhibited excellent UV-blocking ability, with transmittance values close to 0%, confirming their potential as UV-barrier packaging for light-sensitive foods. This feature is particularly relevant for oxidizable foods, as UV radiation accelerates lipid oxidation and nutrient and flavor degradation [25].

In the visible region (350–800 nm), Z/G/NCC films showed high transmittance, whereas the addition of AL progressively reduced visible light transmittance due to its unsaturated structure [20]. The barrier effect increased proportionally with AL concentration. Although reduced transparency may be considered a drawback, since it limits product visibility, it can be advantageous for products containing visible light–sensitive compounds, such as riboflavin, myoglobin, and hemoglobin, which absorb light above 400 nm and are prone to quality deterioration under light exposure [26]. A similar behavior was reported by Xia, Ma, Ma, Yao, Xu, Ji, and Zhang [20] for cellulose-based films incorporated with AL.

Table 3 presents the results of the films’ physical, mechanical, and thermal properties. Compared to the Z/G/CNC control film, adding AL led to a significant increase in thickness, from 0.18 mm to a range of 0.19 to 0.26 mm. This increase may be attributed to the higher solid content and the molecular structure of AL, which could interfere with the packing density of the polymer matrix [16].

Regarding mechanical properties, AL incorporation resulted in a reduction in tensile strength (TS) and Young’s modulus (E), accompanied by an increase in elongation at break (EB), as shown in Table 3. The decrease in TS may be associated with the formation of discontinuities and heterogeneities in the film matrix caused by the presence of AL, as evidenced by the SEM micrographs in Figure 2.

Despite the decline in mechanical strength, the increased EB suggests that AL may act as a plasticizing agent, enhancing the material’s flexibility. The reduction in Young’s modulus reinforces this interpretation, indicating lower stiffness and greater ductility, consistent with the observed deformation behavior. A study by AAhammed et al. [27] reported TS values ranging from 7.04 MPa to 10.99 MPa for Z/G films. In the present study, using the same zein/gelatin ratio but incorporating CNC, the films exhibited significantly higher TS values, reaching up to 61.5 MPa, approximately 6 to 9 times greater than previously reported. This substantial reinforcement highlights CNC’s structuring and reinforcing role within the polymer matrix, promoting greater cohesion and integrity. Moreover, even after the addition of AL, the functionalized films maintained high mechanical performance, indicating that CNC could mitigate the adverse effects of AL on the film structure. Thus, the developed films demonstrate excellent performance and are technically suitable for food packaging applications, where materials with strong mechanical properties are required to ensure product protection and integrity during storage and transport.

**Figure 4:**
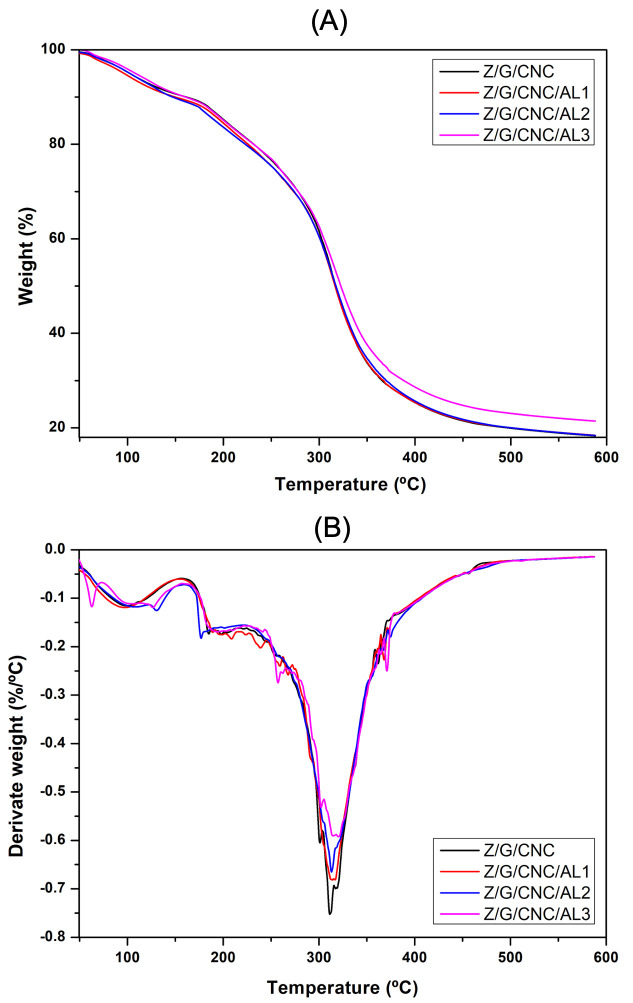
TG (**A**) and DTG (**B**) curves of zein/gelatin/cellulose nanocrystal-based nanocomposite films functionalized with alizarin.

The thermogravimetric (TG) and derivative thermogravimetric (DTG) curves of AL-incorporated films are shown in Figure 4. All films exhibited three distinct weight-loss stages. The first stage (<100 °C) corresponds to the evaporation of residual solvents, such as acetic acid, absorbed by the polymer matrix [1]. The second stage (150–250 °C) is associated with glycerol evaporation and protein decomposition [28], consistent with the glycerol degradation behavior reported by Ezati and Rhim [12]. The third stage (250–400 °C) involves a marked mass reduction due to AL degradation within the polymer matrix [29] and the thermal decomposition of CNC [30]. This significant weight loss is mainly attributed to sample carbonization and the breakdown of thermally stable structural units in the polymer matrix [31].

The onset degradation temperature (Tonset) of the films was not significantly affected by AL incorporation, indicating that the dye did not compromise the initial thermal stability of the material. Minor variations in Tonset may be attributed to the dispersion of AL within the polymer matrix or to specific interactions between its functional groups and the polymers. In contrast, the maximum degradation temperature (Tmax) increased with the addition of AL, particularly at the highest tested concentration (3%), suggesting that AL may enhance the thermal resistance of the films. In this study, the films exhibited higher thermal stability, with both the onset (Tonset) and maximum degradation (Tmax) temperatures exceeding those reported by Xia, Ma, Ma, Yao, Xu, Ji, and Zhang [20] for cellulose-based films containing AL, which showed Tonset around 150 °C and Tmax between 280 and 310 °C.

**Figure 5:**
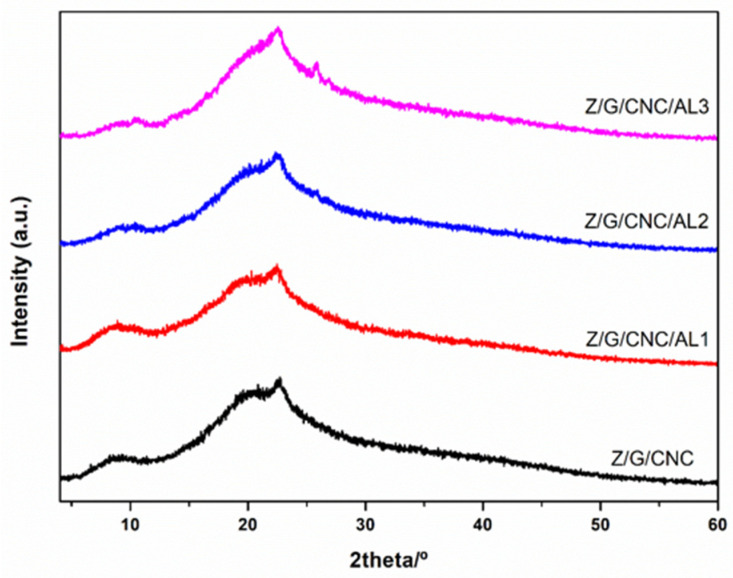
XRD patterns of smart films based on zein (Z), gelatin (G), and cellulose nanocrystals (CNC) functionalized with alizarin (AL).

Figure 5 shows the XRD patterns of films incorporated with AL. The diffraction profiles were similar, exhibiting three characteristic peaks at approximately 8.6°, 20.0°, and 22.6°. The peaks at 8.6° and 20.0° are associated with the protein-based biopolymer matrix, whereas the diffraction angle at 22.6° corresponds to the cellulose structure [32]. These patterns indicate a low degree of crystallinity, which is directly related to the use of acetic acid as a solvent during film preparation [33]. This effect can be attributed to two main mechanisms: (i) the ability of acetic acid to disrupt the intrinsic crystalline structures of both Z and G, and (ii) its inhibitory effect on recrystallization during the formation of the composite material [34]. The incorporation of AL did not significantly affect the crystallinity of the films, as no differences were observed between the XRD patterns of AL-containing and AL-free films. A similar result was reported by Zou, Chen, Dou, Zhu, Zhao, Wang, Wang, and Xia [24], who found that adding AL to PCL nanofibrous films did not alter the diffraction patterns compared to neat PCL films.

**Figure 6:**
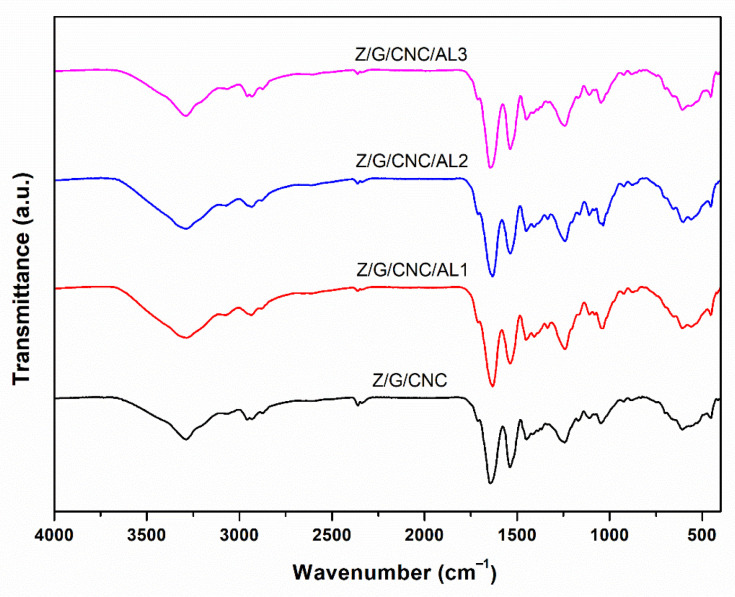
XRD patterns of smart films based on zein (Z), gelatin (G), and cellulose nanocrystals (CNC) functionalized with alizarin (AL).

The FTIR spectra (Figure 6) of the zein/gelatin/cellulose nanocrystal (Z/G/CNC) films, with and without AL, displayed similar overall profiles, indicating that the incorporation of AL did not significantly alter the molecular structure of the polymeric matrix. A broad absorption band at 3292 cm^−1^, assigned to O–H stretching of cellulose nanocrystals [35], and weaker C–H stretching at 2937 cm^−1^ were observed in all samples. Characteristic cellulose bands at 1246 and 1037 cm^−1^, corresponding to C–O stretching of saccharide units [36], remained unchanged, confirming that the polysaccharide component was not structurally affected by the incorporation of AL. The typical protein-related bands were also evident: the amide I band at ~1634 cm^−1^ (C=O stretching of carbonyl and –COO^−^ groups), amide II at ~1537 cm^−1^ (N–H bending coupled with C–N stretching), and amide III at ~1242 cm^−1^ (C–N stretching and in-plane N–H bending) [1]. Notably, films containing AL exhibited slightly more intense bands at ~1640 and ~1540 cm^−1^, with the intensity increasing in the order AL1 < AL2 < AL3, which is characteristic of aromatic ring vibrations [37]. These subtle changes provide clear evidence of the successful incorporation of AL while confirming that its addition did not significantly disrupt the original polymeric network.

**Figure 7:**
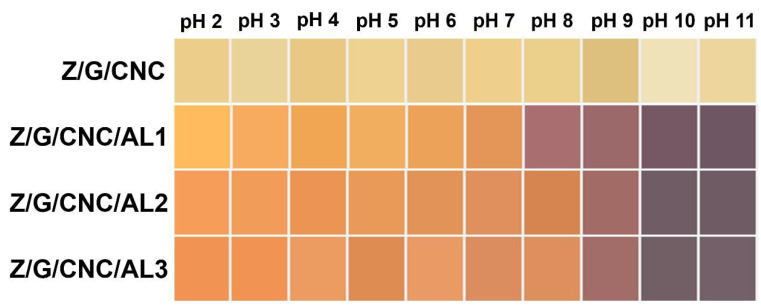
Color changes at different pH ranges of smart films based on zein (Z), gelatin (G), and cellulose nanocrystals (CNC) functionalized with alizarin (AL).

AL imparts to the films the ability to change color according to the acidity or alkalinity of the medium, making them promising candidates for use as pH sensors in smart food packaging [19]. As shown in Figure 7, the films displayed an orange hue from pH 2 to 8, turning lilac at pH 9 and purple between pH 10 and 11. Similar behavior was reported by He et al. [38] for PCL nanofibers incorporated with AL, which exhibited a color transition from yellow to purple within the same pH range observed in this study.

The Z/G/CNC/AL1 film showed a more abrupt color change when exposed to pH 8, suggesting that lower AL concentrations promote greater homogeneity within the polymer matrix (as also evidenced in Figure 2) and, consequently, higher sensitivity and responsiveness to pH variations.

The color transition was visible to the naked eye, highlighting the material’s sensitivity and reinforcing the potential of AL-functionalized films as biodegradable optical sensors for real-time monitoring. These findings are consistent with previous reports describing similar pH-dependent color changes in AL-based films [39].

**Figure 8:**
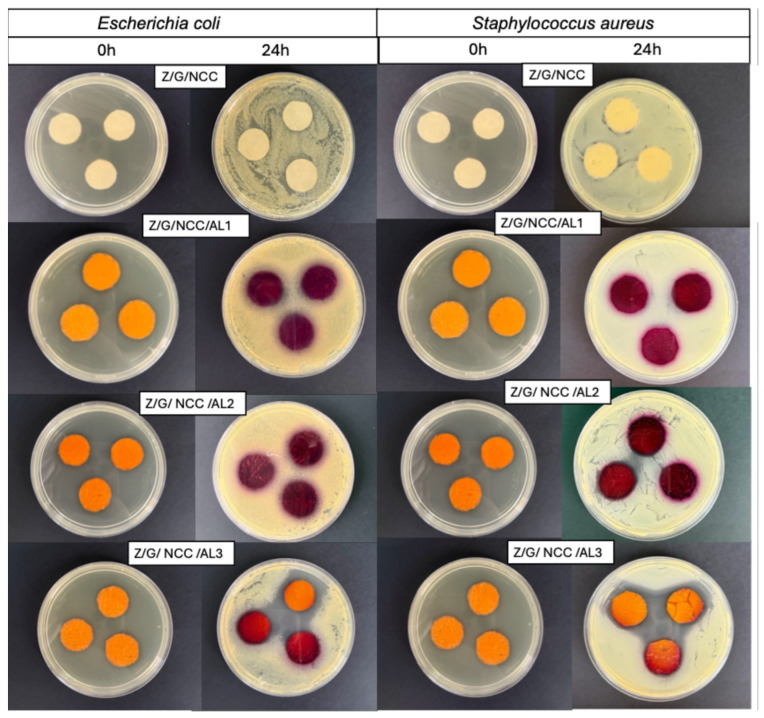
Microbial response of smart films based on zein (Z), gelatin (G), and cellulose nanocrystals (CNC) functionalized with alizarin (AL) to *E. coli* and *S. aureus* strains upon direct contact in trypticase soy agar medium.

As shown in Figure 8, Z/G/CNC films incorporated with AL exhibited a color change from orange to purple after 24 h of direct contact with *E. coli* and S. aureus. This response is attributed to the deamination of proteins during bacterial growth, which increases the alkalinity of the medium, resulting in the color shift of the film [16].

The film containing the highest AL concentration (3%) exhibited an inhibition halo of 16.35 mm and 19.42 mm (Figure 8) to *E. coli* and S. aureus, respectively. Although the antimicrobial activity is a desirable property, the inhibition of bacterial growth reduced the colorimetric response. Therefore, films with lower AL concentrations may be more suitable for use as smart indicators. The antimicrobial potential of AL has also been previously reported [40].

### 3.2. Application of Smart Films for Monitoring Fish Fillet Quality

**Figure 9:**
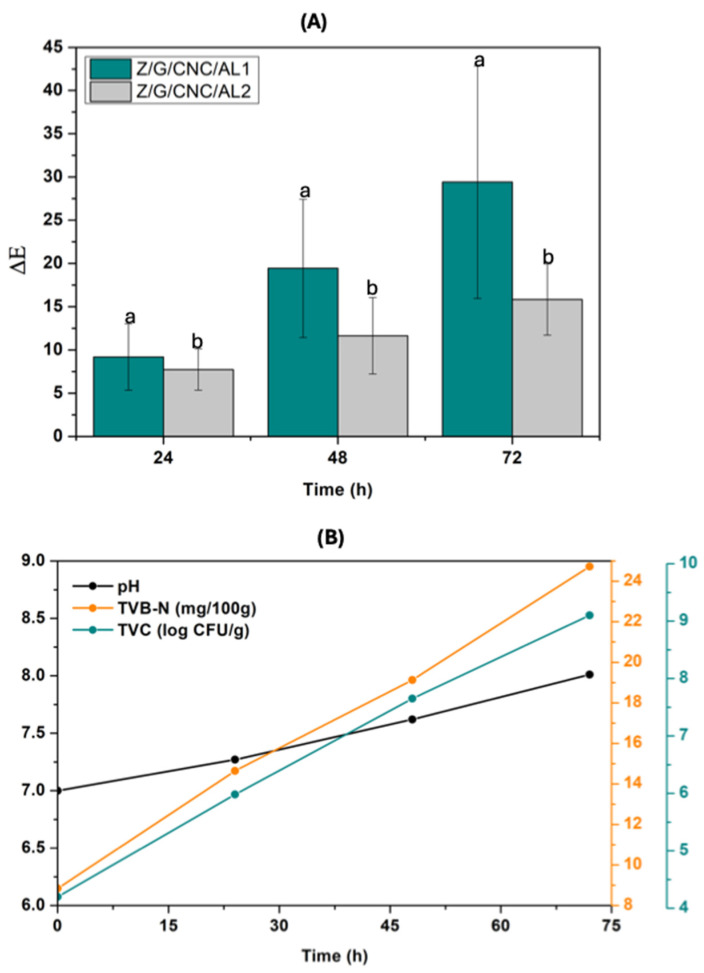
Application of smart films for freshness monitoring of fish. (**A**) Perceptive color difference (ΔE) and (**B**) pH, volatile base nitrogen measurement (TVB-N), and aerobic plate count (TVC). Bars within the same time point with different letters differ significantly according to Student’s *t*-test (*p* < 0.05).

**Figure 10:**
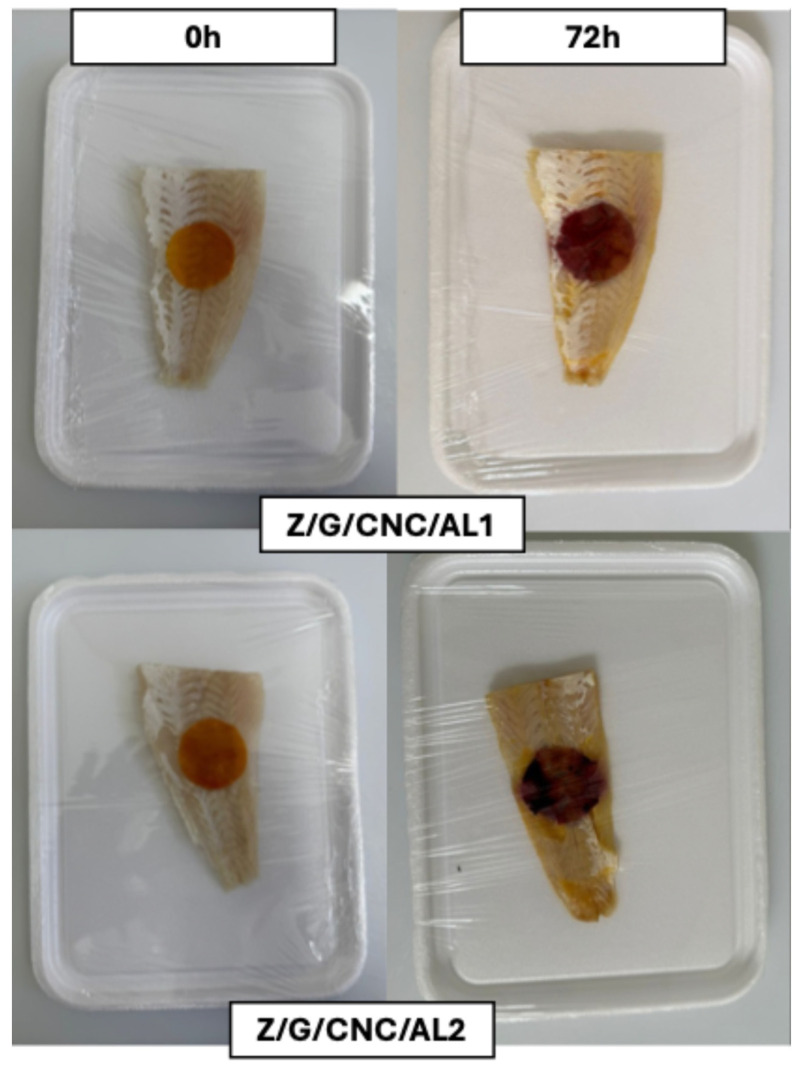
Color change of the smart films in response to fish spoilage during 72 h of storage at 25 °C.

The Z/G/CNC/AL1 and Z/G/CNC/AL2 films were applied to monitor the quality of hake fillets (Figure 9). The ΔE values of the films progressively increased during storage after 24 h, reaching ΔE > 3, indicating a visually perceptible color change (Figure 9A). Initially, the films exhibited an orange hue, which darkened to deep orange after 24 h and shifted to purplish tones as spoilage progressed, reaching 72 h (Figure 10).

Concurrently, the hake fillets showed an increase in pH (from 7.00 to 8.14), total volatile elemental nitrogen (TVB-N) levels (from 8.84 to 24.73 mg/100 g), and total bacterial counts (from 4.2 to 9.01 log CFU/g) during 72 h of storage (Figure 9B). These increases were attributed to the metabolic activity of spoilage microorganisms, which promote protein degradation and the release of volatile nitrogenous compounds [41]. Considering that the rejection limit for TVB-N in fish flesh is 20 mg/100 g [18]. After 72 h of storage (Figure 9B), the fillets became unsuitable for consumption, coinciding with the films’ color change to purplish tones. These findings demonstrate the potential of Z/G/CNC/AL films as effective visual indicators for monitoring fish quality during storage.

## 4. Conclusions

The zein/gelatin/cellulose nanocrystal (Z/G/CNC) films functionalized with alizarin (AL) demonstrated excellent potential as smart packaging for fish. Alizarin incorporation enhanced thermal resistance, UV-blocking capacity, and pH sensitivity, while maintaining sufficient mechanical integrity for packaging applications. Z/G/CNC/AL films showed a distinct color transition from orange to purple after 24 h of direct contact with *E. coli* and *S. aureus* and displayed clear, visually perceptible color changes correlated with spoilage indicators in fish (pH and TVB-N). These findings demonstrate that Z/G/CNC/AL films are sustainable and effective alternatives for smart packaging, contributing to improved food safety and waste reduction.

## Figures and Tables

**Table 1:** Water-related properties of nanocomposite films based on zein (Z), gelatin (G), and cellulose nanocrystals (CNC) functionalized with alizarin (AL).

Treatments	WS (%)	WVP (g·mm/kPa-h-m^2^)	WCA (°)
Z/G/CNC	80.25 ± 5.36 ^a^	2.76 ± 1.21 ^b^	67.39 ± 4.58 ^c^
Z/G/CNC/AL1	63.09 ± 2.40 ^b^	3.97 ± 1.13 ^a^	97.84 ± 8.56 ^a^
Z/G/CNC/AL2	64.09 ± 3.20 ^b^	3.05 ± 1.63 ^ab^	81.58 ± 8.04 ^b^
Z/G/CNC/AL3	60.73 ± 1.14 ^c^	3.15 ± 2.44 ^a^	72.48 ± 5.77 ^c^

Values in the same column followed by at least one common letter (or not followed by any letter) are insignificantly different according to Tukey’s test (*p* < 0.05).

**Table 2:** Chromaticity of nanocomposite films based on zein (Z), gelatin (G), and cellulose nanocrystals (CNC) functionalized with alizarin (AL).

Treatments	L	h (°)	C (*)	ΔE
Z/G/CNC	87.39 ± 1.80 ^a^	93.91 ± 0.87 ^a^	19.87 ± 5.60 ^d^	-
Z/G/CNC/AL1	71.30 ± 4.16 ^b^	76.31 ± 4.87 ^b^	76.45 ± 2.25 ^a^	86.09 ± 2.71 ^a^
Z/G/CNC/AL2	61.12 ± 1.32 ^c^	60.28 ± 1.99 ^c^	69.92 ± 3.11 ^b^	81.58 ± 2.80 ^b^
Z/G/CNC/AL3	57.03 ± 2.12 ^d^	55.01 ± 2.27 ^d^	66.48 ± 3.21 ^c^	79.58 ± 2.37 ^c^

Values in the same column followed by at least one common letter (or not followed by any letter) are insignificantly different according to Tukey’s test (*p* < 0.05).

**Table 3:** Thermo-mechanical properties of nanocomposite films based on zein (Z), gelatin (G), and cellulose nanocrystals (CNC) functionalized with alizarin (AL).

Treatments	Thickness (mm)	Tensile Strength (MPa)	Elongation at Break (%)	Young’s Modulus (%)	T_onset_ (°C)	T_max_ (°C)
Z/G/CNC	0.19 ± 0.02 ^c^	61.5 ± 13.72 ^a^	9.75 ± 2.01 ^c^	52.61 ± 12.02 ^a^	195.54	316.12
Z/G/CNC/AL1	0.18 ± 0.04 ^c^	36.78 ± 5.45 ^c^	20.17 ± 2.75 ^b^	19.81 ± 6.71 ^c^	193.31	317.24
Z/G/CNC/AL2	0.22 ± 0.04 ^b^	39.81 ± 5.23 ^b^	18.64 ± 6.88 ^b^	32.16 ± 5.90 ^b^	185.05	317.24
Z/G/CNC/AL3	0.26 ± 0.04 ^a^	24.56 ± 12.72 ^d^	23.23 ± 3.37 ^a^	18.04 ± 3.96 ^c^	194.43	324.23

Values in the same column followed by at least one common letter (or not followed by any letter) are insignificantly different according to Tukey’s test (*p* < 0.05).

## Data Availability

The data presented in this study are available on request from the corresponding author. The data are not publicly available due to privacy restrictions.
